# A Review and Clinical Practice Guideline for Health Professionals Working With Indigenous and Culturally and Linguistically Diverse (CALD) Populations During COVID-19

**DOI:** 10.3389/fpubh.2021.584000

**Published:** 2021-06-25

**Authors:** Rony Kayrouz, Carlie Schofield, Olav Nielssen, Eyal Karin, Lauren Staples, Nickolai Titov

**Affiliations:** ^1^MindSpot Clinic, Macquarie University, Sydney, NSW, Australia; ^2^eCentreClinic, Department of Psychology, Macquarie University, Sydney, NSW, Australia

**Keywords:** ethnicity, clinical practice guideline, indigenous, health professionals, health communication

## Abstract

**Background:** As the rates of infection and mortality from COVID-19 have been higher in minority groups, the communication of health information in a way that is understood and accepted is of particular importance.

**Aims:** To provide health professionals with a clinical practice guideline for clear and culturally sensitive communication of health information about COVID-19 to people of Indigenous and culturally and linguistically diverse (CALD) backgrounds.

**Assessment of Guideline Options:** The authors conducted a review of the literature on health communication, and the guidelines were developed with particular reference to the SPIKES protocol of “breaking bad news” in oncology and the use of the DSM-5 Cultural Formulation Interview (CFI).

**Actionable Recommendations:** The guideline combines two approaches, the Cultural Formulation Interview, developed for DSM-5, and the SPIKES protocol used for delivering “bad news” in oncology. The combined CFI-SPIKES protocol is a six-step clinical practice guideline that includes the following: (1) Set up (S) the interview; (2) Determine how the patient perceives the problem (P) using the Cultural Formulation Interview (CFI) to elicit the patient's cultural perception of the problem; (3) Obtain an invitation (I) from the patient to receive a diagnosis; (4) Provide the patient knowledge (K) of diagnosis in a non-technical way; (5) Address the patient's emotional reaction (E) to diagnosis; and (6) Provide the patient a summary (S) of healthcare and treatment.

**Conclusions and Relevance:** This article presents guidelines for assessing the cultural dimensions of patients' understanding of COVID-19 and delivering diagnostic and treatment recommendations in ways that are culturally safe and responsive, such as: (a) suspending the clinician's own cultural biases to understand the explanatory models and cultural values of their CALD or Indigenous patients; (b) encouraging the use of interpreters or cultural brokers to ensure that that the message is delivered in a way that the patient can understand; and (c) encouraging CALD or Indigenous patient to take an active part in the solution and treatment adherence, to minimize transmission of COVID-19 in CALD and Indigenous communities.

## Introduction

Severe acute respiratory syndrome coronavirus 2 (SARS-CoV-2, also referred to as COVID-19) continues to spread worldwide. When preparing this report there were more than 154 million confirmed cases of COVID-19 and more than 3.2 million deaths (1). The only way of controlling the spread of COVID-19 is to reduce the rate of transmission through a combination of quarantine measures, social distancing, and vaccination. To do so requires community-wide understanding and adherence to hygiene and public health recommendations (2).

There has been considerable variation between countries and regions, such as, China, South Korea, Europe, Africa, and the US, in the recommendations and measures adopted to enforce social distancing, isolation and quarantine (3). Inconsistent messaging has resulted in confusion and delays in initiating measures to control what has proven to be a highly infectious virus. Contradictory health advice can have a particular effect on vulnerable populations such as Indigenous and Culturally and Linguistically Diverse (CALD) groups living in Western countries such as Australia, Europe, USA, and the UK, who are often suspicious of mainstream services, may have less access to health advice that they trust and also often experience overcrowding and multigenerational households. Studies have found that cultural differences in explanatory models cultural values, preferences for doctor-patient relationships, the perception of racism and cultural biases, and linguistic barriers can have the effect of reinforcing stigma, increasing mistrust, and reducing access to medical treatment in CALD and Indigenous populations (4). Hence, unclear or contradictory communication that reinforces stigma and increases mistrust may contribute to people from Indigenous and CALD communities being less likely to adopt the recommended social distancing and isolation measures, not accessing testing for COVID-19, not cooperating with contact tracing, and not trusting vaccinations (5).

Hence an important task for health professionals during the COVID-19 pandemic is to ensure that health communication of COVID-19 to Indigenous and CALD people is clear and culturally sensitive. This communication can be achieved by considering historical and cultural perspectives of infection, cultural interpretations and preferences for receiving medical advice. Health professionals need to reflect on their communication and ensure their advice is understood. The use of interpreters or cultural brokers can be crucial to address linguistic barriers and improve the patients' understanding of healthcare (6), as in many cultures, it is polite to agree, even when the information is not understood.

Patient engagement and informed decision making are critical in all health and mental-health communication, irrespective of ethnicity (7–9). Research has found that providing reliable information in a way that is readily understood and encouraging patients to generate a list of questions regarding their health care helps address some communication barriers and, increases patient engagement and trust in health services for CALD populations (8, 10). Stigma needs to be addressed and trust built with patients who experience shame in response to receiving a diagnosis (11–13).

Patients from Indigenous and CALD backgrounds need to experience health services as culturally safe to reduce stigma and build trust with health services. Patients from Indigenous and CALD backgrounds frequently report finding health service staff to be unwelcoming and unfriendly (14, 15). Cultural safety requires that clinicians engage in the process of self-reflection about patients' rights and the power dynamics of a patient-clinician relationship. This process of reflection requires that the clinician understand how their cultural values and biases can affect the patient's sense of safety and being understood (14).

One protocol designed to increase a patient's sense of cultural safety is the Royal College of Physicians and Surgeons of Canada (RCPSC) cultural safety guidance for clinicians during the COVID-19 pandemic (16). The guideline provides practical advice for clinicians working with Indigenous populations in Canada to ensure culturally safe assessment and treatment of patients for COVID-19. Some examples are the option for self-swabbing, social distancing may not be possible because of overcrowding, and creating safe locations for testing that do not resemble institutional settings that are welcoming to family members and connected to the land. Further, the protocol provides excellent overarching guidelines for clinicians that include: (1) awareness of past traumatic experiences; (2) Build relationships that create trust; (3) results and gathered information is owed by the patient; and (4) Consider resources and affordability when discussing solutions. However, the RCPSC cultural safety guidance does not provide clinicians with specific questions to increase their understanding of the patient's perception of COVID-19, such as the patient's explanatory model of COVID-19, past treatment experiences, past and current help-seeking, coping, and treatment preferences, and the family's or community's explanatory model of COVID-19 and treatment preferences for the patient. Furthermore, the guideline did not provide suggestions about dealing with a patient's emotional reactions to COVID-19 diagnosis or if patients have divergent beliefs about COVID-19 and may not want a diagnosis or to be tested for COVID-19.

Two protocols that addressed the gaps of the RCPSC guideline and were designed to improve clinician's cultural competence, which would, in turn, increase patient cultural safety, engagement, and informed decision making are the SPIKES protocol (17, 18) and the Cultural Formulation Interview (CFI) (19). Both support clinicians in assessing the cultural dimensions of a patient's understanding of their diagnosis and deliver diagnostic and treatment recommendations in culturally responsive and safe ways. This skill is particularly critical when the patient's explanatory model of their illness diverges from the clinician's explanatory model.

The SPIKES protocol was initially developed to help clinicians respond to patients with cancer and their relatives who did not want the cancer diagnosis disclosed as culturally they saw it as detrimental to the patient's health. The CFI protocol was developed to help clinicians respond to all patients with mental health conditions exploring their culturally based explanatory models of mental illness, coping, help-seeking, and treatment preferences. The limitation of the SPIKES model is that it did not provide enough detailed guidelines for exploring the patient's perception of the problem, something the CFI does very well. The CFI model does not explicitly guide how to discuss the diagnosis with a patient or their emotional reaction. Combining the CFI and SPIKES protocol could address these gaps and would not need to be used with patients with similar explanatory models and treatment preferences, who are eager to know their diagnosis.

We suggest that combining these protocols for patients who report cultural beliefs and practices of any health condition that diverges from the dominant medical model may increase a patient's sense of cultural safety and engagement. This divergence is likely to be more significant with new and emerging conditions that are not well-understood, such as COVID-19. There is a great diversity of beliefs around the causes (e.g., 5G, work of the devil, government control) and treatment available for COVID-19. The combined protocol provides a framework for clinicians to respond to this diversity in a culturally safe and responsive manner while maintaining ethical obligations of providing the best available information and evidence about COVID-19 and vaccinations.

Consistent with this proposal, there is emerging evidence indicating that the CFI and SPIKES protocols improve culturally sensitive communication of health information. Preliminary evidence suggests that the CFI (20, 21) and SPIKES (22, 23) can improve clinical communication by enhancing practitioner-patient rapport, allowing the clinician to elicit patients' cultural views on what caused their symptoms, and help patients to access their cultural practices and resources as part of their healthcare solution in oncology and transcultural psychiatry, respectively. The further rationale for combining the SPIKES and CFI protocols are 3-fold. First, a study that provided external assessment by a psychologist and senior clinician of SPIKES protocol adherence found 5th-year medical students performed poorly in finding out “what the patient knows” (P) and “what the patient wants to know” (I) (24). Second, the similarity between COVID-19 and cancer in the requirement for the “breaking of bad news” means the SPIKES protocol can alert the health professional to the need to communicate in an empathic manner. Thirdly and most importantly, similar to receiving a mental health diagnosis, a COVID-19 diagnosis for a CALD or Indigenous patient may not result in cooperation with medical advice because of stigma, mistrust in health services, and other structural barriers to adherence (e.g., discrimination, racism), which can be minimized if the health professional uses the CFI protocol to communicate in a culturally safe and responsive manner.

Hence, this paper aims to follow the AGREE reporting checklist (25) and provide health professionals with a clinical practice guideline, that is, the CFI-SPIKES protocol, that respects the cultural diversity of opinions and treatment preferences of patients by the culturally sensitive communication of health information about COVID-19, while presenting the best available evidence about COVID-19 to patients of Indigenous and CALD backgrounds.

## Assessment of Guideline Option

A search of peer-reviewed journals on cultural formulation interview (CFI) and SPIKES protocol was conducted up to May 2020. Key search terms used in all electronic databases included a combination of the following: (1) For CFI, “cultural formulation interview” and (2) For SPIKES, “SPIKES” and “cancer” and “breaking bad news.” The following electronic databases were searched for SPIKES and CFI: (a) PubMed and (b) PsycINFO. Complete searching key terms in clinical trials and humans to identify empirical studies on the feasibility of CFI and SPIKES protocols (see Table 1).

**Table 1:** Inclusion table.

**Inclusion**	
Study design	Quality Ratings Scheme for Studies and Other Evidence (26) 1 Properly powered and conducted randomized clinical trial; systematic review with meta-analysis 2 Well-designed controlled trial without randomization; prospective comparative cohort trial 3 Case-control studies; retrospective cohort study 4 Case series with or without intervention; cross-sectional study 5 Opinion of respected authorities; case reports
Participants	Clinicians and Patients (18 years and over)
Intervention	Cultural Formulation Interview or SPIKES protocol
Comparator or Control	All single group open trials and comparative studies of CFI and SPIKES including waitlist control

**Figure 1:**
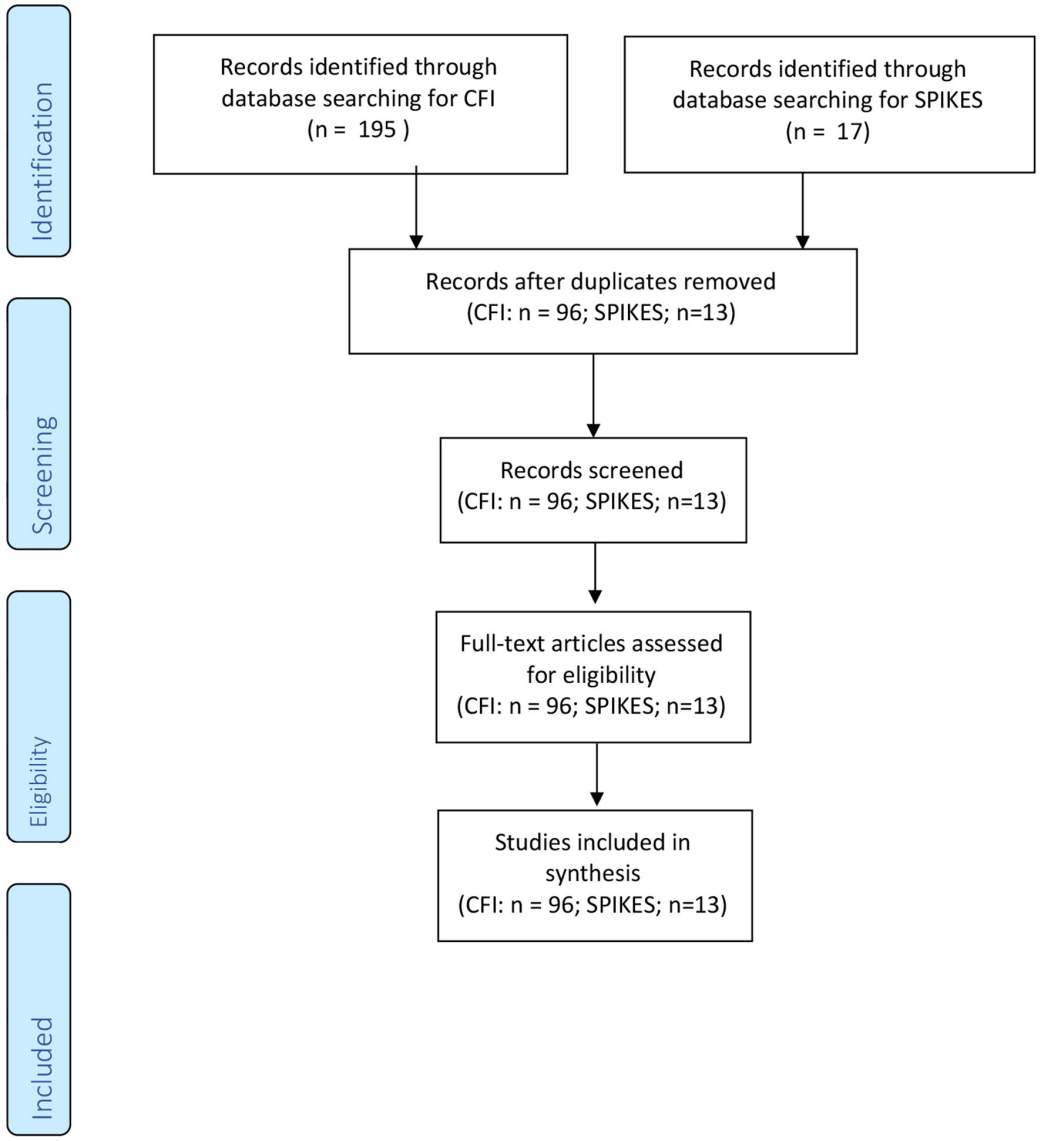
Review of the feasibility of the SPIKES and CFI protocol.

**Figure 2:**
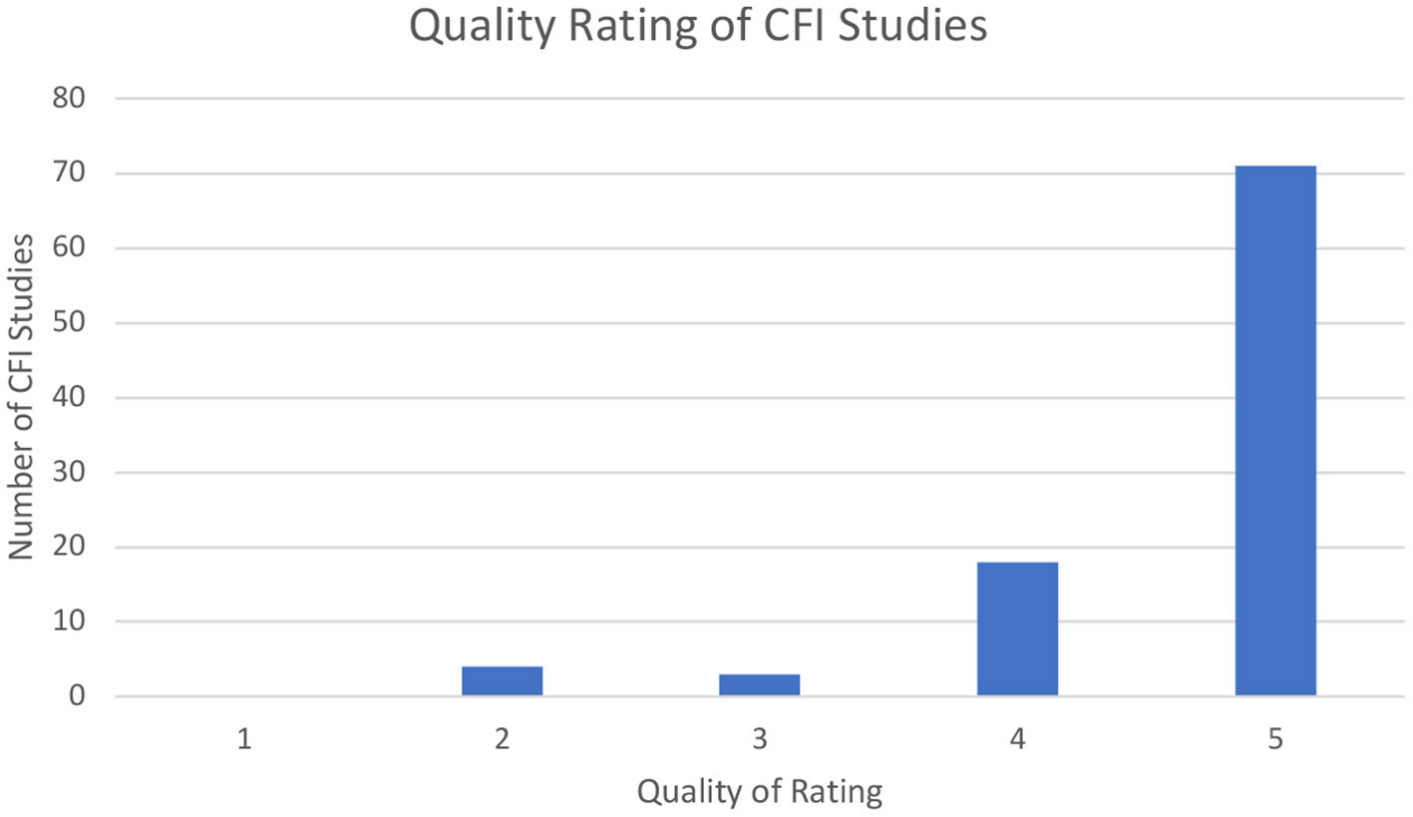
Quality rating of CFI studies.

**Figure 3:**
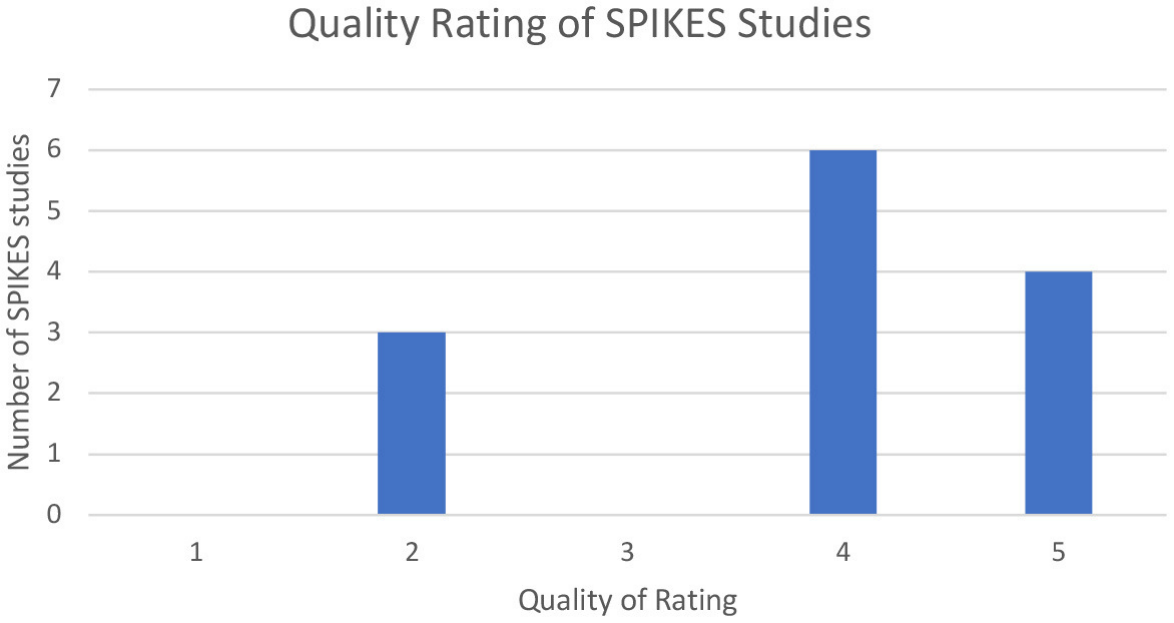
Quality rating of SPIKES studies.

For the CFI and SPIKES, our search of the databases generated 195 and 17 articles, respectively. After duplicate removal and assessing titles and abstracts, 96 for CFI and 13 for SPIKES were eligible (see Figure 1). For CFI, four articles received a rating of 2 (i.e., well-designed controlled trial without randomization or prospective comparative cohort trial), three articles received a rating of 3 (i.e., case-control studies or retrospective cohort study), 18 articles received a rating of 4 (i.e., case series with or without intervention; cross-sectional study), 71 articles received a rating of 5 (i.e., opinion of respected authorities or case reports) (see Figure 2). For SPIKES, three articles received a rating of 2, none received a rating of 3, six articles received a rating of 4, and four articles received a rating of 5 (see Figure 3). For CFI [χ^2^(4, 96) = 184.7, *p* < 0.001] and SPIKES [χ^2^(4, 13) = 10.5, *p* = 0.033], the proportion of high-quality studies with a rating of 1–3 was significantly lower than the proportion of low-quality studies with a rating of 3–5.

### Details of Eligible Studies

Details of eligible studies that rated 2 for CFI and SPIKES are reported in Tables 2, 3, respectively. For CFI, all four (100%) studies (*n* = 758) were published in English. Two (50%) studies were performed in six countries (i.e., Canada, India, Kenya, Netherlands, Peru, and the USA). For SPIKES, all three (100%) studies (*n* = 232) were published in English. Two (67%) studies were performed in Canada and one (33%) in France.

**Table 2:** Summary of feasibility studies of CFI with a quality of evidence rating of 2.

**References**	**Design**			**Sample**	**Results**	
	**Conditions *(n)***	**Treatment duration and mode of delivery**	**Design, Primary measure/s**	**(*N*, Age, *F*%, Country)**	**Completion rate (%)**	**Post-treatment and follow-up effects**
Aggarwal et al. (27)	Clinicians (14)	Online training and case discussion and role-play training on CFI	(SGOT, four time-points, Evidence-Based Practice Attitude Scale)	Clinicians from Northeastern Psychiatric Center(13, 46.31, 77%, USA)	Clinicians (93%)	Overall attitude to adoption of CFI amongst clinicians did not change at 10-month follow-up.
Hinton et al. (28)	Montreal (33) New Delhi (67) Pune (36) Nairobi (30) Lima (34) Netherlands (30) USA (91)	CFI	(Comparative cohort study, CFI questionnaire and semi-structured interview)	Patients from Local Clinics(321, 34, 45%, Canada, USA, Netherlands, Kenya, Peru and India)	Patients (100%)	All sites (1 = Agree) Feasibility >1 Acceptability >1 Clinical Utility >1 No follow-up or effect size calculated
Mills et al. (29)	Psychiatry Residents (30)	1-h didactic session on CFI	(SGOT, Cultural Competence Assessment Tool)	Psychiatry Residents Program(30, 26–30, 50%, USA)	Residents (73%)	Cultural Knowledge, Non-Verbal Communication showed significant improvement. No follow-up or effect size calculated
Lewis-Fernandez et al. (30)	Patients (318) Clinicians (75)	CFI	(Comparative cohort study, CFI questionnaire)	Patients and Clinicians outpatient services(393, 41.4, 50%, Canada, USA, Netherlands, Kenya, Peru and India)	Patients (100%) Clinicians (100%)	All sites for Patients (1 = Agree) Feasibility, Acceptability and Clinical Utility > 1 All sites for Clinicians (1 = Agree) Feasibility, Acceptability and Clinical Utility: 0.75–0.93 No follow-up or effect size calculated

*CFI, Cultural Formulation Interview; SGOT, Single group open trial*.

**Table 3:** Summary of feasibility studies of SPIKES with a quality of evidence rating of 2.

**References**	**Design**			**Sample**	**Results**	
	**Conditions (n)**	**Treatment duration and mode of delivery**	**Design, Primary measure/s**	**Arab sample(*N*, Age, *F*%, Country)**	**Completion rates (%)**	**Post-treatment and Follow-up**
Bonnaud-Antignac et al. (24)	Medical Students (108)	Assess training of SPIKES course using three sessions, S1 Lecture, S2 Video-taped simulated interviews and S3– Feedback from senior physician	(SGOT, self-reported assessment of competence by student)	5th Year Medical Students(108, 28.1, 69%, France)	S1 (76%) S2 (63%) S3 (77%)	S3 > S2 > S1 (increased competence in breaking news, use of communication techniques, and self-knowledge.
Papadakos et al. (31)	Healthcare providers (64)	A blended multi-professional communications program, online theoretical learning and reflective practice	(SGOT, self-reported assessment of competence based on participants' motivational beliefs).	Healthcare Providers (64, 33.6, 68%, Canada)	Healthcare providers (98%)	Statistically significant increase in self-perceived competence in breaking bad news, disclosing incidents, and responding to challenging behavior
Sherwood et al. (32)	Students (47) Physicians (13)	Small physician-led groups taught breaking bad news using the SPIKES framework	(SGOT, self-reported assessment of competence)	Students and Physicians (60, ns, ns, Canada)	Students (89%) Physicians (77%)	In pre-session, 13% (6/45) of students indicated comfort with the skill of breaking bad news, compared with that in post-session with 81% (34/42)

*F%, percentage of Females in sample; ns, not specified; SGOT, Single group open trial*.

### Demographic Characteristics

Demographic characteristics of the participants are shown in Tables 2, 3. For CFI, across the four studies (*n* = 758), 89 clinicians (11.7%), 30 psychiatry residents (4.0%), and 639 patients (84.3%), were evaluated. Clinicians provided mental health care for an average of 14.5 years, and psychiatry residents' training ranged from post-graduate years 1–4. Patients' average years of education was 10.6. Patients who participated in the studies were coming from Canada (10.3%), India (32.1%), Kenya (9.3%), Netherlands (9.3%), Peru (10.6%), and the USA (28.4%). Two of the included studies in the current review had large sample sizes (i.e., 321 and 393 participants), and the other two had small sample sizes (i.e., 13 and 30). The mean age of all the participants was 37.4, of which 55.5% were female.

For SPIKES, of the three studies, one study evaluated medical students, one study evaluated healthcare providers, and one study evaluated students and physicians. All three studies included medium sample sizes (i.e., 108, 64, 60). The mean age of all the participants was 30.9, of which 68.5% were female.

### Delivery Characteristics

For CFI, two of the four CFI protocols (50%) were delivered in a combined individual and group format, one in a group and one as an individual. Two of the four studies (50%) were comparative cohort studies, and the other two (50%) were single group within-subject studies. All (100%) of the didactic training of CFI was delivered by health professionals.

For SPIKES, all three studies (100%). SPIKES protocol was delivered in a group format. All of the studies were single group within-subject studies and delivered by health professionals.

### Outcome Measures

Different outcome measures were used to assess the feasibility, acceptability, and clinical utility of CFI.

Two (50%) of the studies used a standardized questionnaire about the acceptability, feasibility, and clinical utility of CFI for patients and clinicians. The other two studies (50%) used standardized measures, Cultural Competence Assessment Tool and Evidence-Based Practice Attitude Scale, to assess the clinicians' cultural competence and attitudes toward adopting the CFI protocol, respectively. For SPIKES, all studies used self-reported assessment of cultural competence.

### Acceptability, Feasibility, and Clinical Utility

For CFI, one study demonstrated a significant improvement in cultural competence (6.0%) due to the CFI training. Specifically, significant improvements occurred in a clinician's cultural knowledge (10.8%) and non-verbal communication (11.8%) (29). Another study found that the clinician's attitudes for adopting the CFI protocol were maintained at 10-month follow-up (27). Two studies found that the CFI was deemed acceptable, feasible, and clinically useful by clinicians and patients from Canada, India, Kenya, Netherlands, Peru, or the USA (28, 30). Hinton and colleagues conducted a cross-site comparison between Pune, New Delhi, and Nairobi patients that found that Nairobi patients reported higher acceptability, feasibility, and clinical utility of the CFI (28). Completion rates for CFI ranged from 73 to 100%.

For SPIKES, all three studies found that clinician's reported greater perceived competence in delivering bad news (66, 60, and 37%). Completion rates ranged from 63 to 100%.

## Actionable Recommendations

The guideline combines two approaches, the Cultural Formulation Interview, developed for DSM-5, and the SPIKES protocol used for delivering “bad news” in oncology. The combined CFI-SPIKES protocol includes the following six steps,

1. Set up the interview (S);
2. Determine how the patient perceives the problem using the Cultural Formulation Interview (CFI) to elicit the patient's cultural perception of the problem, coping and treatment preferences (P);
3. Obtain an invitation from the patient to receive a diagnosis (I);
4. Provide the patient knowledge of diagnosis in a non-technical way (K);
5. Address the patient's emotional reaction to diagnosis (E);
6. Provide the patient a summary of healthcare and treatment (S).

**1. Take time to set up (S) the interview and get to know the patient before communicating COVID-19 diagnosis and quarantine measures**.

Ensure the room or meeting space (e.g., nursing homes, private practices, consulting room in hospitals or make-shift hospitals, or telehealth consultation) is set up, so there is privacy and no interruptions. Also, use interpreters or cultural brokers, by telephone or video if necessary, when a patient's main spoken language is not the same as the health professionals. In this stage, the clinician is getting to know the patient and looking to increase the patient's sense of cultural safety by building rapport and trust. It should be noted that many Indigenous and CALD populations operate from a high context culture (33), where direct communication of diagnosis is sometimes not welcomed, making a patient feel culturally unsafe and may rupture rapport and trust. Consequently, talking around the context and impact of the diagnosis could be more critical for some patients. Moreover, the direct communication of diagnosis without consideration of context may trigger previous negative experiences of being spoken at, misunderstood, judged, and discriminated. Hence, the first step of the health professional could be to establish a respectful and trusting relationship with the patient, where the patient experiences cultural safety before they can be open to the communication of health information. This trust-building can be facilitated by taking some time to get to know a little about the patient's background, level of education, life experience, previous experience of health care, and attitudes toward health in general before communicating the diagnosis of COVID-19 and information about quarantine measures and treatment of COVID-19.

**2. Take time to understand how the patient perceives (P) or understands COVID-19 and quarantine measures?**

The Indigenous or CALD patient is seen as the expert on their culture, and health professionals are invited to suspend their beliefs about COVID-19 and listen to how the patient understands the virus and its actions and implications. The DSM-5 Cultural Formulation Interview (CFI) (19) provides a helpful framework:

a) How does the patient culturally define the problem of COVID-19 and associated quarantine measures? How does the patient understand social distancing and quarantine measures?
b) How does the patient explain the causes of COVID-19? This can be potentially the most challenging part of the conversation, particularly when the patient may have an explanation for the illness that diverges from the scientific model health professionals may hold. Take time to listen and be open and respectful to the different ways patients will explain the causes of COVID-19, including 5G networks, spiritual attributions such as the work of evil spirits, or a disturbance in harmony between the land, the spirit, and the people.
c) How does the patient's cultural identity make having a COVID-19 diagnosis better or worse? Will a patient be stigmatized and outcasted from the community because of the diagnosis or receive support from their community?
d) What are the patient's cultural ways of coping and seeking help because of COVID-19?
e) What are the barriers to getting help?
f) What cultural factors affect current help-seeking and treatment preferences? Suppose the patient attributes the cause of COVID-19 to supernatural processes or as a disturbance of balance/harmony. In that case, treatment preferences may involve ritual, and religious practices from spiritual healers and elders to dispel evil spirits and reestablish harmony and balance. Moreover, ask patients what their culture thinks about social distancing and quarantine measures of isolation? Ask them to tell you in the past what actions their communities have taken when dealing with people with infectious diseases. If there are measures that differ from the current recommendations, provide a rationale about how the lack of social distancing and quarantine for 14 days may place their elders and others in their respective communities at very high risk.

**3. Obtain the patient's invitation (I) to disclose the diagnosis of COVID-19**.

This step challenges the assumption that all patients need to know if they have COVID-19. Nevertheless, a respectful invitation about whether patients want to know if they have the diagnosis of COVID-19 needs to be asked. In the cases where the patient says they do not want to know, then a rationale needs to be provided about how them not knowing their diagnosis and practicing social distancing will put others, especially by placing the elders in their communities, at a greater risk of contracting COVID-19.

**4. Provide the patient knowledge (K) about the COVID-19 diagnosis in a non-technical way and in their native language**.

It is essential to use non-technical language that a patient can understand when communicating COVID-19 diagnosis, quarantine measures, and treatment options. Provide a detailed explanation of the symptoms and quarantine measures associated with COVID-19 rather than using medical terms or abbreviations. Provide handouts or direct patients, cultural brokers, and interpreters to translated online resources about COVID-19. Concerning treatment options, provide the best available evidence in a non-technical way that addresses any concerns the patient may have about treatment.

**5. Address the patient's emotional (E) reactions to the diagnosis of COVID-19 via empathy and referral to culturally appropriate health services if needed.**

People from Indigenous or CALD communities may experience shame and guilt. The shame may be associated with beliefs that a patient is being punished by God or ancestors for wrongdoings or that the balance in the harmony and oneness of the community was attributed to some individual actions against the community. Health professionals will need to normalize and provide empathy and refer to respective culturally appropriate or indigenous health services.

**6. Provide the patient a summary (S) about the diagnosis, quarantine measures, and treatment**.

Provide a summary of diagnosis, the necessity of quarantine measures, and cooperation with contact tracing and where to obtain treatment. Then check in with the patient how well they have understood the diagnosis, quarantine measures, and treatment plan if necessary. In this step, you could consider the following:

a. **Allow patients to generate a list of questions regarding their treatment and health care:** Patient engagement and informed decision-making are critical in health and mental-health communication, irrespective of ethnicity (7–9). Research has found that providing good information and supporting patients to generate a list of questions regarding their health care address some of these communication barriers, increasing patient engagement levels across some CALD populations (8, 10).
b. **Social Distancing Guidelines**: Indigenous and CALD communities tend to be more collectivist cultures and may struggle with the advice to adhere to social distancing. Consequently, a very clear rationale about the need for social distancing in terms of protecting their cultural group and others needs to be communicated to increase the likelihood of adherence. In addition, due to overcrowding in some Indigenous and CALD communities, social distancing and quarantine measures may not be possible.
c. **Develop Support Plan**: Finally, help people from Indigenous and CALD communities develop a support plan with people so they know that they can call on these people if they run out of essential items or if they need emotional support.

## Discussion

The aim of this paper was to provide health professionals with a clinical practice guideline, that is, the CFI-SPIKES protocol, that promotes culturally sensitive communication of health information about COVID-19 to patients of Indigenous and CALD backgrounds. There is some evidence supporting the use of SPIKES and CFI protocols to improve clinicians' competence in delivering bad news [e.g., (32)] and communicating to people from different cultures (29). Separately the CFI (30) and SPIKES (34, 35) have been found to be feasible and acceptable by patients. Although the combined CFI-SPIKES protocol has only been evaluated as a case study (36), in the context of communicating information about COVID-19, the combination provides health professionals with a guideline that delivers the bad news of a diagnosis of COVID-19 in a culturally sensitive manner and reminds the health professional to find out more about “what the client culturally knows” (P) about COVID-19 and “what the client wants to culturally know” (I) about COVID-19 using the CFI.

Using this guideline can help health professionals address the barriers of stigma, mistrust, and language and become more culturally competent in health communication in the following ways. First, by helping health professionals suspend their perspective and explanatory models to understand the explanatory models and cultural values of their CALD or Indigenous patients. Second, by encouraging the use of interpreters or cultural brokers to address linguistic and cultural barriers and ensure that the message is delivered in a way that the patient can understand. Third, by promoting patient engagement and informed decision-making so that CALD or Indigenous patients can participate in the solution and treatment adherence.

A limitation of the CFI, SPIKES, and CFI-SPIKES protocols is that randomized control trials (RCT) evaluating the efficacy of these protocols in terms of increasing cultural competency, patient understanding, and treatment adherence have not been conducted. Thus, this clinical practice guideline recommendations are not based on direct evidence but extrapolation from studies of the acceptability and utility of the tools in other contexts. Nevertheless, the CFI and SPIKES protocols are likely to increase the cultural responsiveness of clinicians in their communication of health information, understanding of the patient's explanatory models, coping and treatment preferences, ensuring patients feel culturally safe and understood. Future research should evaluate how the CFI-SPIKES protocol impacts stigma, mistrust in health services, patient's sense of cultural safety, communication of health information, and treatment engagement and adherence, which will inform the future updating of the CFI-SPIKES guideline.

This article presents guidelines for assessing the cultural dimensions of patient's understanding of COVID-19 and delivering diagnostic and treatment recommendations in culturally safe and responsive ways. The pandemic has increased the importance of health communication to Indigenous and CALD patients. If they feel respected and understood by health professionals and are made active collaborators in the solution of their healthcare for COVID-19, there is a greater chance that they will trust the information and adhere to health advice. The adoption of these guidelines may help minimize the spread of COVID-19 amongst CALD and Indigenous populations.

## Author Contributions

RK conceived and designed the study, conducted a literature review, interpreted the data, and drafted the manuscript. NT, CS, EK, LS, and ON reviewed the design, analysis, interpretation of data, and the manuscript. All authors have contributed to, read, and approved the manuscript.

## Conflict of Interest

The authors declare that the research was conducted in the absence of any commercial or financial relationships that could be construed as a potential conflict of interest.
